# Dementia, infections and vaccines: 30 years of controversy

**DOI:** 10.1007/s40520-023-02409-8

**Published:** 2023-05-09

**Authors:** Fiona Ecarnot, Virginia Boccardi, Andrea Calcagno, Claudio Franceschi, Tamas Fülop, Ruth F. Itzhaki, Jean-Pierre Michel, Francesco Panza, Innocenzo Rainero, Vincenzo Solfrizzi, Andrea Ticinesi, Nicola Veronese, Stefania Maggi

**Affiliations:** 1 EA3920, University of Franche-Comté, 25000 Besancon, France; 2 Institute of Gerontology and Geriatrics, Department of Medicine and Surgery, University of Perugia, Santa Maria Della Misericordia Hospital, Piazzale Gambuli 1, 06132 Perugia, Italy; 3 Unit of Infectious Diseases, Department of Medical Sciences, University of Turin, Turin, Italy; 4 Laboratory of Systems Medicine of Healthy Aging, Institute of Biology and Biomedicine and Institute of Information Technology, Mathematics and Mechanics, Department of Applied Mathematics, N. I. Lobachevsky State University, Nizhny Novgorod, Russia; 5 Department of Medical and Surgical Sciences, Alma Mater Studiorum, University of Bologna, Bologna, Italy; 6 Department of Medicine, Geriatrics Division, Research Center on Aging, Faculty of Medicine and Health Sciences, University of Sherbrooke, Sherbrooke, QC J1H 5N4 Canada; 7 Institute of Population Ageing, University of Oxford and Faculty of Life Sciences, University of Manchester, Manchester, UK; 8 University Hospitals of Geneva, Geneva, Switzerland; 9 Unit of Research Methodology and Data Sciences for Population Health, National Institute of Gastroenterology “Saverio de Bellis”, Research Hospital, Castellana Grotte, Bari, Italy; 10 Dipartimento Interdisciplinare di Medicina, Clinica Medica e Geriatria “Cesare Frugoni”, University of Bari Aldo Moro, Bari, Italy; 11 Dementia Center, Department of Neuroscience “Rita Levi Montalcini”, University of Torino, Turin, Italy; 12 Department of Medicine and Surgery, University of Parma, Parma, Italy; 13 Geriatric-Rehabilitation Department, Azienda Ospedaliero-Universitaria di Parma, Parma, Italy; 14 Geriatrics Section, Department of Internal Medicine, University of Palermo, Palermo, Italy; 15 National Research Council, Neuroscience Institute, Aging Branch, Padua, Italy; 16 Department of Cardiology, University Hospital Besancon, 3-8 Boulevard Fleming, 25000 Besancon, France

**Keywords:** Dementia, Alzheimer’s disease, Infection, Virus, Bacteria

## Abstract

This paper reports the proceedings of a virtual meeting convened by the European Interdisciplinary Council on Ageing (EICA), to discuss the involvement of infectious disorders in the pathogenesis of dementia and neurological disorders leading to dementia. We recap how our view of the infectious etiology of dementia has changed over the last 30 years in light of emerging evidence, and we present evidence in support of the implication of infection in dementia, notably Alzheimer’s disease (AD). The bacteria and viruses thought to be responsible for neuroinflammation and neurological damage are reviewed. We then review the genetic basis for neuroinflammation and dementia, highlighting the genes that are currently the focus of investigation as potential targets for therapy. Next, we describe the antimicrobial hypothesis of dementia, notably the intriguing possibility that amyloid beta may itself possess antimicrobial properties. We further describe the clinical relevance of the gut–brain axis in dementia, the mechanisms by which infection can move from the intestine to the brain, and recent findings regarding dysbiosis patterns in patients with AD. We review the involvement of specific pathogens in neurological disorders, i.e. SARS-CoV-2, human immunodeficiency virus (HIV), herpes simplex virus type 1 (HSV1), and influenza. Finally, we look at the role of vaccination to prevent dementia. In conclusion, there is a large body of evidence supporting the involvement of various infectious pathogens in the pathogenesis of dementia, but large-scale studies with long-term follow-up are needed to elucidate the role that infection may play, especially before subclinical or clinical disease is present.

## Introduction

The possibility of an infectious etiology for dementia, and in particular Alzheimer’s disease (AD), has repeatedly been postulated over the past 30 years. There is now a large body of evidence to show that infiltration of the brain by pathogens may act as a trigger or a cofactor for AD, by one or several mechanisms. Firstly, pathogens may directly weaken the blood–brain barrier, and cross into the central nervous system, ultimately causing neurological damage by eliciting neuroinflammation. Alternatively, pathogens may cross the intestinal barrier, reach the vascular circulation, and then cross the blood brain barrier (BBB), or cause low-grade chronic inflammation and subsequently neuroinflammation from the periphery. In the case of several pathogens such as herpes simplex virus type 1 (HSV1) and *C. pneumoniae*, entry is thought to be either through the olfactory route or in the case of HSV1, from the trigeminal ganglia, not the BBB. The European Interdisciplinary Council for Aging convened a 2-day virtual meeting on 24–25 November 2022, to review the state of the evidence on the link between infection and neurological disorders and dementia. We present here the Executive Summary of the proceedings of this meeting. Firstly, we briefly summarize the literature relating to the infectious theory of dementia, from the first hypotheses proposed more than 30 years ago, to the latest results supporting the direct involvement of infectious agents in the pathophysiology of dementia. We next describe the genetic basis for neuroinflammation and dementia, examine the hypothesis of an “anti-microbial” effect, and discuss the connection between the intestinal microbiota and dementia, or the so-called gut–brain axis. Next, we examine the link between specific infectious agents and neurological disorders (SARS-CoV-2, human immunodeficiency virus, herpes simplex virus type 1, influenza), and the possible protective role of vaccination against such pathogens in preventing or mitigating cognitive decline. Finally, considering the unanswered questions highlighted by current research, we suggest some avenues for future research in elucidating the link between infection and dementia. Without a doubt, improving uptake of vaccination against key vaccine-preventable diseases is an attractive and inexpensive means to lower the burden of infectious disease, and its attendant consequences, in older populations [1–4].

## Infectious-related dementia: 30 years of controversy

Dementia is the fifth non-communicable cause of death globally, and the second cause in high-income countries [5]. It significantly increases in-hospital mortality among older subjects [6]. However, at present, there is limited, if any disease-modifying treatment, with the result that the burden of dementia, and particularly AD is likely to continue growing.

The first efforts to identify a causal connection between neurodegenerative disorders and infection started in the early 1980s, culminating in the publication in 2016 of an editorial providing evidence of a causal role of pathogens in AD [7]. This was the foundation of the microbial hypothesis of AD [7, 8], presenting strong links supporting the role of microbes in AD. We should nevertheless note that already Alois Alzheimer and very shortly after, Oskar Fischer raised the possibility of an infectious origin behind their observations concerning the patient considered as the first subject with this type of dementia.

The microbial hypothesis of AD postulates that aging and weakening of the BBB and the immune system due to infection or age-related processes, together enable infection of healthy brain by viruses, bacteria or other pathogens (fungi, parasites). The molecular components of these pathogens, such as DNA/RNA, proteins, enzymes or lipopolysaccharides (LPS) may contribute to chronic low-grade neuroinflammation, resulting in enhanced production of amyloid-β (Aβ) and aggregation of hyperphosphorylated tau protein, culminating in neurodegeneration and cognitive dysfunction [9].

A number of single-taxon viral infections have been associated with AD, such as Herpes simplex virus (HSV) (types 1 and 2), HHV6, varicella zoster virus (VZV), human immunodeficiency virus (HIV), Epstein–Barr virus (EBV), cytomegalovirus (CMV) or hepatitis C virus (HCV) [10]. Following infection, these viruses can enter a latent phase and potentially become reactivated in the event of immune impairment. Indeed, 90% of adults have been infected with at least one form of herpes virus [10]. The most robust evidence relates to the implication of HSV-1, with evidence for a causal role of this pathogen in AD (see specific section below). There is also evidence of a role for human cytomegalovirus in AD, with an association first proposed in 1979 [11], and CMV seropositivity associated with a twofold increase in the risk of AD and with faster cognitive decline in a population of 849 older subjects [12]. Furthermore, anti-CMV IgG levels were significantly associated with faster cognitive decline [13], and CMV antibody levels with neurofibrillary tangles (NFTs) [14]. VZV has also been implicated in AD, with a retrospective cohort study of 846 patients and 2538 controls reporting that the incidence of dementia during 5 years of follow-up was 2.97-fold greater in those experiencing herpes zoster ophthalmicus compared to controls [15].

In the same way, various single-taxon bacteria have also been associated with AD, including *Chlamydia pneumoniae*, *Escherichia coli, Helicobacter pylori, and Porphyromonas gingivalis*. In a study of 19 AD patients and 19 controls, *C. pneumoniae* was identified in brain areas with typical AD-related neuropathology in 17 out of the 19 AD patients, whereas identical brain areas were PCR negative in 18/19 controls [16]. Brain areas infected included those connected to olfaction, such as the amygdala entorhinal cortex, as well as the hippocampus proper and temporal and frontal cortices. All cell types including glia (microglia and astroglia), neurons, and endothelia were infected to some extent [16]. In another seminal study, mice infected with *C. pneumoniae* were shown to have Aβ deposits in the central nervous system (CNS) at 2 months post-infection [17]. Finally, a meta-analysis of 25 case–control studies showed a pooled odds ratio of 5.66 (95% confidence interval (CI) 1.83–17.51) for occurrence of AD when there was detectable evidence of *C. pneumoniae* infection [18]. Periodontal bacteria have been reported to be implicated in AD. The main oral bacterium implicated in AD is *Porphyromonas gingivalis *[19]*.* In serological studies, an AD group exhibited elevated levels of antibodies against several bacteria [20, 21]. Another serological study on seven periodontal bacterial species reported significantly increased antibody levels against *Fusobacterium nucleatum* and *Prevotella intermedia* in AD [22]. Finally, co-infection with *H. pylori* and periodontal pathogens was shown to interact synergistically, altering the risk of AD and all-cause dementia [9, 23].

Taken together, the evidence supporting the infectious burden hypothesis of dementia postulates that the single- or multi-taxon infection may promote chronic inflammation, increased production, and reduced clearance of Aβ and/or phosphorylated tau, ultimately engendering the features characteristic of AD. If ongoing trials of antimicrobial agents to mitigate or prevent AD were to show positive clinical results, this would represent a major leap forward in our understanding of AD neuropathogenesis.

## Genes, neuroinflammation and dementia

The term “dementia” relates to a complex syndrome that results from a lifetime of interaction between genetic, lifestyle, environmental and age-related factors. AD is the most common cause of dementia, but several other diseases may also cause dementia.

Heritability shows great variability in the different forms of dementia, ranging from 5 to 50%, with some having a very low grade of heritability, whereas others, such as frontotemporal dementia (FTD), having high (45–50%) heritability [24].Dementia is of monogenic origin in few patients, whereas for the largest group, the disease is explained by the complex interactions of many risk factors interacting with environmental factors, and recent thinking indicates that AD is a polygenic or oligogenic disorder [25]. Very recently, the European Alzheimer & Dementia Biobank (EADB) consortium identified 75 independent loci for AD; 42 were new genetic risk factors for dementia, opening up new perspectives for gene-specific treatments, and personalized medicine [26].

Technological progress in genomics analysis has helped to reveal the huge range of genetic risk factors potentially implicated in AD [27], and there is a compelling need to move away from the concept of a single genetic factor, towards a convergent pathway that leads to dementia [28]. One such pathway involves neuroinflammation, which is generally defined as the activation of the brain’s innate immune system in response to an inflammatory challenge. It is characterized by acute molecular changes within the brain. In addition, low grade, chronic inflammatory mechanisms have been implicated in a variety of neurodegenerative conditions, including AD, FTD, Parkinson's disease (PD), and Amyotrophic Lateral Sclerosis (ALS). It remains a matter of debate whether neuroinflammation causes or accelerates neurodegenerative disease.

Neuroinflammation in AD and PD is reflected by morphological changes in glial cells, including astrocytes and the microglia. The use of animal models helped to elucidate the mechanisms of neuroinflammation. A mouse model of ALS in which the gene encoding mutant superoxide dismutase-1 (mSOD1) was expressed, yielded a severe phenotype of motor neuron death. The deletion of the transgene from microglia produced an unexpected prolongation of life span without altering the timing of disease onset [29]. Another important gene is *C9orf72*, whose deficiency promotes a change in the homeostatic signature in microglia and a transition to an inflammatory state characterized by an enhanced type I IFN signature. C9orf72-depleted microglia were shown to trigger age-dependent neuronal defects leading to altered learning and memory behaviours in mice [30]. In a study of another gene, namely *TREM2*, single nucleus RNA sequencing of microglia from patients carrying the *TREM2* R47H mutation and from sporadic AD patients revealed the existence of a new subset of microglia, designated as amyloid-responsive microglia, specifically involved in dementia [31].

It is a frequently held dogma that there is a differentiation between central and peripheral mechanisms of neuroinflammation. However, studies of patients with ALS for example have found both central and peripheral immune dysfunction [32], featuring immune cell infiltration into the CNS, dysregulated peripheral immune cell counts, and altered cytokine production [32].

A poorly studied aspect of neuroinflammation is the gender effect. Auto-immune diseases are more common in women (who account for up to 80% of cases), and women typically have enhanced immunoreactivity compared to men. Sex differences in neuroinflammatory response may be particularly relevant among older adults, given that menopause potentiates low-grade, chronic inflammation. A post-mortem study showed that microglial activation in AD pathogenesis was disproportionately higher in females than in males [33], while data from the Alzheimer’s Disease Neuroimaging Initiative (ADNI) showed that soluble tumor necrosis factor receptor 2 (sTNFR2) cerebrospinal fluid (CSF) concentrations were related to poorer cognition in women only [34].

Several genes have been implicated in neuroinflammation. Among these, genetic variants and polymorphisms of the *TREM2* gene have been found to be associated with AD, FTD, and possibly PD. Additionally, *TREM2* genetics has shown unmistakably that microglia dysfunction or infiltrating myeloid cells could be a primary rather than a reactive contributor to neurodegeneration [35, 36]. Closely related to *TREM2* is the *CD33* gene, expressed mainly in the microglia. Genome-wide association studies (GWAS) support a role of *CD33* in AD. This receptor inhibits the uptake and clearance of the beta peptide in the microglial cells, thereby promoting neuroinflammation and neurodegeneration. The most robust evidence for a genetic component of AD relates to the human apolipoprotein E (apoE), a 299-amino acid secreted glycoprotein binding cholesterol and phospholipids, with three common isoforms (APOE ε2, APOE ε3, and APOE ε4). Carriage of the ε4 allele of the *APOE* gene is an established risk factor for AD and other forms of dementia (dementia with Lewy bodies (DLB), FTD). In the CNS, ApoE is involved in various pathways including synaptic function, blood–brain barrier integrity, cytoskeletal assembly, mitochondrial integrity and dendritic morphology and function. Several lines of evidence suggest that ApoE plays an important role in modifying systemic and brain inflammatory responses (for review, see [37]). Finally, the *PGRN* (progranulin) gene, and its active protein progranulin, can be measured directly in the plasma. The *PGRN* gene is mutated in a subset of patients with FTD and interacts with various inflammatory mechanisms. Progranulin binds to the receptor for TNFR and modifies both acute inflammatory response and apoptotic mechanisms [24, 38].

In recent years, a new concept has been gaining traction as the final step in a long process of degeneration, namely the mechanism called efferocytosis, or the removal of apoptotic cells by phagocytes. The effective clearance of apoptotic cells is essential for maintaining CNS homeostasis and restoring homeostasis after injury. In most cases of physiological apoptotic cell death, efferocytosis prevents inflammation and other pathological conditions. However, when apoptotic cells are not effectively cleared, destruction of the integrity of the apoptotic cell membrane, leakage of intracellular contents, and secondary necrosis may ensue. Investigating the functional impact of AD-associated variants and genes in microglia is essential for elucidating disease risk mechanisms and developing effective therapeutic approaches.

Finally, no discussion of the genetic mechanisms involved in AD would be complete without a discussion of epigenetics, namely how the environment influences genetic characteristics. It is established that the main epigenetic processes, including DNA methylation, histone modifications or noncoding RNAs, are closely associated with neurodegenerative diseases. Dysregulated epigenetics on microglia-related genes may affect the function of microglia, resulting in neuroinflammation and neuronal damage. In an investigation of DNA methylation levels of 7 key immunologic-related genes in the peripheral blood from 222 participants (101 AD, 72 mild cognitive impairment, and 49 non-cognitively impaired controls), Li et al. reported that methylation levels were altered in AD, and statistical models that included methylation biomarkers improved prediction of AD [39].

With the growing body of evidence attesting to the genetic mechanisms involved in neuroinflammation and neurodegenerative disease, it is natural to raise the question of whether neuroinflammation can be targeted for treatment, to prevent or delay disease onset. However, despite a strong therapeutic rationale, studies to date that have investigated compounds with anti-inflammatory properties to modulate neuroinflammatory processes in dementia have been unsuccessful. There are several possible reasons for this. Firstly, it is conceivable that study design issues, including the lack of diagnostic accuracy and biomarkers for target population identification and proof of mechanism, may partially explain the negative outcomes. Second, there is a need to better specify the characteristics of the different forms of dementia, and genetic classification may open perspectives for specific therapeutics. Future perspectives include studies of *TREM2* and *CD33*, which have emerged as potential therapeutic targets in AD. Monoclonal antibodies against the CD33 and TREM2 proteins have entered clinical trials and may reduce neuroinflammation in the AD brain. Elsewhere, Hinderer et al. evaluated adeno-associated viral vector-mediated delivery of *GRN* into the CSF in animal models, to compensate for progranulin deficit and restore function. After vector delivery into the lateral cerebral ventricles, they observed increased progranulin levels, reduced lysosomal lipofuscin deposits, normalized lysosomal enzymatic activity, and reduced microgliosis [40].

In summary, many studies have investigated the genetic architecture of diseases that cause dementia, showing a complex interplay between several different genes. Genes regulating neuroinflammation are paramount in dementia and may cause and/or accelerate long-term neurodegeneration, playing a central role in the very early stages of disease development. Knowledge of the different genetic causes of dementia and their associated phenotypes is essential to propose appropriate genetic diagnosis to patients and to plan a personalized therapeutic strategy.

## The antimicrobial hypothesis of Alzheimer disease

AD was first recognized based on the accumulation of neurofibrillary tangles and deposits of amyloid beta protein in the brain, and these features have become the pathological hallmarks of dementia. This gave rise to what is commonly called the amyloid cascade hypothesis of AD, and central to this theory is the amyloid beta protein, which has receptors on many cell types and can stimulate receptors to enter cells by signalling of pro-inflammatory transcription, moving to activation of microglia and destruction of neurons. This theory focused predominantly on amyloid beta protein as the main culprit for AD. However, as outlined above, it has emerged in recent years that neuroinflammation is also a very important contributing factor, with growing evidence of an association between the immune system and AD.

However, a key problem that has undermined the validity of this paradigm is the failure of many clinical trials targeting the amyloid beta protein. While pathologically, amyloid-targeted drugs may decrease the burden of amyloid protein, there has been no meaningful clinical effect on the disease [41]. In addition, emerging evidence suggests that amyloid beta has also numerous biological roles, some of which are protective, and that these functions are exerted in a hormetic/antagonistic pleiotropy manner, i.e. neuroprotective at low concentrations and pathological at high concentrations [42]. Further arguments that contradict the amyloid cascade hypothesis are the fact that amyloid beta is found in many older adults who do not have dementia, while many demented patients have no amyloid beta. Recent data examining the change in peripheral innate immune response during the progression of cognitive disease has shown that there are distinct phenotypic and functional changes in monocyte and macrophage populations with increasing severity of cognitive impairment [43]. There is also activation of the immune system in the brain linked to neuroinflammation via the activation of microglia and astrocytes. Indeed, when there is a state of stress in the organism, there may be activation and passage of molecules from the periphery to the brain, through the meninges, across the blood–brain barrier, or via the choroid plexus. Immune cellular players in AD, including cell types such as astrocytes, microglia, macrophages, monocytes, endothelial cells, and T-cells, are all activated to various degrees, rendering appreciation of their respective roles very difficult [44].

It is now known that infection can increase the activation of the peripheral immune system, which will generate a systemic inflammatory state, and may cross the blood–brain barrier to cause neuroinflammation and the resultant pathological hallmarks. In this regard, it is important to distinguish between acute and chronic infection. Acute encephalitis is a reaction that is meant to eliminate pathogens and clear out the aggression via activation of adaptive immune system. Nevertheless, these initial infections may become chronic/latent and reactivate during decades and lead ultimately to neurodegeneration. Therefore, neurodegeneration is chronic, and not focused on elimination of the pathogens, but on trying to mitigate the effect, namely the production of amyloid beta protein, the spread of amyloid beta, and the resultant characteristics plaques, as well as the neurofibrillary tangles. Viruses and bacteria, and the microbiota–gut–brain axis have all been extensively studied as initiators of the infectious processes, either directly or indirectly, that perturb all levels of intra and extracellular functions and culminate decades after the initial infection in clinical signs of cognitive decline.

The large body of intriguing findings raised the question of whether amyloid beta might have antimicrobial properties. Antimicrobials may exert their action either by directly killing invasive pathogens, or via immune modulation by penetrating the cells, raising the perspective of numerous possibilities that could be harnessed to stop or decrease viral or bacterial invasion, with a resultant effect on neuroinflammation. Very recent evidence indicates that amyloid peptides display functional roles against microbes not only via antimicrobial function (membrane disruption, protein aggregation or altered protein conformation within the microbe) but also via microbe agglutination function [45]. Amyloid beta peptides can form or initiate biofilm formation, sequestering and neutralizing the microbes. However, chronic activation of this pathway may ultimately exacerbate amyloid beta deposits, leading to progression towards AD [46].

In conclusion, there is very early involvement of the innate immune system in AD, which is a systemic disease with complex interactions between the periphery and the brain. The common pathway is neuroinflammation, and infection is a trigger which, over decades, will initiate and maintain pathways that ultimately culminate in the disease known as AD, manifesting with cognitive decline. However, it might be better not to call this clinical manifestation AD anymore, but rather, chronic brain insufficiency, potentially of multiple origins, all leading to neurodegeneration.

## Clinical relevance of the gut–brain axis in dementia

The gut microbiome is a community of microorganisms, mainly bacteria but also viruses and fungi, symbiotically living with the host in the gut lumen, with increasing loads from the duodenum to the distal part of the colon. With the technological progress of recent decades, there has been a shift away from traditional culture techniques towards metagenomic approaches, including 16S rRNA microbial profiling and shotgun metagenomics, to quantify and identify the fecal microbiota structure and abundance. These techniques enable measurement of biodiversity and abundance of bacterial taxa within a fecal sample, and assessment of inter-individual variability (metadiversity) within fecal samples from a given population [47].

In healthy adults, the gut microbiota comprises around 10 phyla, with most species belonging to 2 phyla (*Bacteroidetes* and *Firmicutes*). Some taxa are highly represented (e.g. *Bacteroides, Prevotella, Alistipes, Eubacterium*), while there are large numbers of minor players with low representation but exhibiting relevant metabolic activity, such as those that can produce short-chain fatty acids (SCFA) (e.g., *Faecalibacterium, Butyrivibrio, Succinivibrio, Ruminococcus*) [48, 49]. Across the lifespan, the gut microbiota is shaped from childhood towards a stable condition that is reached by the age of about 10 years. In healthy adults, the gut microbiota is characterized by stability over time, and a certain resilience to transient perturbations [50]. The wide variability observed in adults depends on environmental factors, e.g. diet, place of life, drug use, exercise, disease, all of which can shape the gut microbiota structure and composition, and affect inter-individual variability [50, 51].

In aging, gut microbiota faces important changes in composition, including reduced biodiversity, increased uniqueness within the population, and alteration of the balance between representation of symbionts and opportunistic pathogens [51]. Furthermore, the aging microbiota is less stable over time and less resilient to stressors such as courses of antibiotic treatment. In a word, gut microbiota during aging is characterized by a tendency towards dysbiosis, that is, a variation in composition with potential harmful consequences for the physiology of the host. Such consequences may include increased permeability of the intestinal barrier, with translocation of bacterial products into the systemic circulation (e.g. LPS, uremic toxins and cytokines), promoting oxidative stress and both systemic and local inflammation [52].

During aging, dysbiosis may be associated with the functional trajectory of aging. A study by Ticinesi et al. showed that the Chao1 index of biodiversity was associated with 2-year survival after discharge in patients hospitalized with extra-intestinal illness, whereby patients with greater diversity of gut microbiota had better survival [53]. The predictive value of dysbiosis for survival and clinical parameters was confirmed in a larger study performed among 907 subjects from the “Osteoporotic Fractures in Men” cohort (aged 78–98 years). In that study, Wilmanski et al. showed that uniqueness score (measuring the difference from the average of the population) was correlated with survival, and the authors also identified correlations between microbiological parameters and clinical parameters of aging e.g. gait speed [54].

It is, therefore, plausible to hypothesize that the gut microbiota structure is implicated in the aging trajectory, whereby those who age successfully may have a preserved gut microbiota structure that establishes cooperative interactions with the host, while those with frailty and/or disability may have more pronounced dysbiosis that reinforces the mechanisms leading to inflammation and disease, promoting frailty and disability [55]. Dementia and cognitive impairment are a major part of frailty syndrome and the mechanisms mediated by the gut microbiota are, therefore, also important for the pathophysiology of dementia. Indeed, it is known that the gut microbiota can establish a connection with several organs outside the gastrointestinal system, and influence their pathophysiology, highlighting the importance of the gut–brain axis.

The gut–brain interactions occur via several mechanisms. First among these is the vagus nerve and enteric nervous system. The vagus nerve regulates gastrointestinal function and motility, and indirectly, gut microbiota composition [56]. The vagus nerve in the gut mucosa also has some afferent vagal endings that express receptors sensing microbial metabolites (e.g., SCFA) and toxins (e.g., LPS) that may be involved in bottom-up vagal signalling [57]. Animal studies have shown that the activation of these endings may cause an imbalance in brain levels of the neurotransmitters involved in dementia (DOPA, glutamine, GABA) [57]. Second, when gut microbiota dysbiosis is established, it leads to production of cytokines and activation of gut immune cells. This can in turn stimulate peripheral inflammation and neuroinflammation in the brain, and it has been demonstrated that gut microbiota dysbiosis is associated with production of cytokines that can cause activation of microglia, inducing deposits of amyloid beta in the brain [58, 59]. Thirdly, gut microbiota dysbiosis induces alterations of gut mucosa permeability, allowing entry into the systemic circulation not only of bacterial toxins like LPS, but also of live microbes themselves, which can activate the immune cells in the circulation, promoting a systemic inflammatory response, which may affect the microglia, leading to microglial activation and amyloid beta deposition [60]. Indeed, studies in mouse models have underlined complex interplay between leaky gut and leaky blood–brain barrier, specifically at the choroid plexus. During intestinal inflammation associated with dysbiosis, high molecular weight molecules, like bacterial LPS, are allowed to enter the systemic circulation, and in those with no brain illness or dementia, the choroid plexus allows HMW molecules like LPS to enter the cerebrospinal fluids. However, once acute intestinal inflammation is established, the permeability of the choroid plexus is reduced, so the CSF is protected against entry of HMW molecules like LPS [61, 62]. In an animal model, choroid plexus barrier closure was found to be associated with mental deficits [63]. Therefore, there is a rationale to hypothesize that alteration of the blood brain barrier at the level of the choroid plexus may have some role in the pathophysiology of dementia, offering attractive perspectives for future research.

Many bacteria in the gut microbiota can produce short-chain fatty acids (SCFAs), which are important physiological mediators for the host. SCFAs can improve insulin sensitivity, stimulate adipose tissue catabolism and modulate inflammation in the host [64]. Recent studies have also shown their role in the brain [65–67]. SCFAs can inhibit cytokine synthesis and microglial activation, promote neuronal repair and regeneration through the CREB/BDNF pathway, and reduce oxidative stress and inhibit amyloid deposition [65–67]. However, despite these predominantly protective functions, once dementia is established, SCFAs may contribute to enhancing rather than inhibiting amyloid deposition [59]. A Japanese study by Ueda et al. showed in a cross-sectional comparison of gut microbiota composition in several groups of subjects (AD, healthy subjects, and subjects with mild cognitive impairment (MCI)), that depletion of the SCFA producer *Faecalibacterium prausnitzii* was an important characteristic of those with AD/MCI [68]. The authors isolated the strains that were markedly depleted in those with AD and MCI vs healthy subjects, and administered these strains as oral probiotics to mice models of AD, and showed that it led to an improvement on cognitive tests [68].

Despite the exciting evidence from animal models supporting the existence of a gut–brain axis in the pathophysiology of dementia, human studies do not mirror the quality of this evidence, because there is a dearth of data. The human studies investigating the role of the gut microbiota in dementia have been hampered by several limitations, such as cross-sectional design with no follow-up or data on lifestyle habits or diet; small samples sizes; few studies from Western countries; and varying definitions of the outcomes used. Nevertheless, despite the limitations of studies published heretofore, there is a consensus that patients with MCI or dementia have a different fecal microbiota composition compared to healthy controls [69]. Secondly, in most, although importantly, not all studies, AD and MCI patients exhibit reduced representation of bacterial taxa producing SCFAs, and increased representation of *Bacteroidetes *[70, 71]*.*

It is reasonable to explore strategies to manipulate the gut microbiota and modulate the gut brain axis. Evidence of the value of probiotics or functional foods is insufficient to conclude in favour of the utility of these interventions as a means to prevent cognitive decline [72, 73]. A more promising target is diet, and a significant body of evidence exists to show that dietary patterns with positive effects on the gut microbiome (such as the Mediterranean dietary pattern) are associated with improved gut microbiota characteristics and cognitive outcomes [74]. Diet, especially the Mediterranean dietary pattern, as well as exercise could be effective strategies to positively modulate gut microbiota composition and obtain improved outcomes in terms of modulation of gut brain axis and prevention of dementia [75]. Indeed, increased dietary intake of food bioactives, including polyphenols found in many foods of vegetal origin that are a relevant part of the Mediterranean dietary pattern, may provide neuroprotection through metabolic mediation of the gut microbiota [76].

In summary, the gut microbiota plays an established role in cognition and pathophysiology of dementia, but most evidence to date stems from pre-clinical studies. The translation of pre-clinical evidence into clinical practice remains uncertain, because there is currently a lack of high-quality human studies in this field. Strategies aimed at maintaining gut microbiota eubiosis are probably effective in preserving cognition, but further clinical research is needed to identify strategies that maintain gut eubiosis, via healthy diet, exercise, and virtuous use of drugs and vaccines.

## Specific pathogens and neurological disorders

### Central nervous system complications of SARS-CoV-2

The severe acute respiratory syndrome coronavirus 2 (SARS-CoV-2) enters the lung cells via the spike proteins, which confer the ability to enter the cells through the ACE2 receptor [77], allowing activation and migration of the virus into the system. The cell-free and macrophage-phagocytosed virus can spread to other organs and infect ACE2-expressing cells at local sites, causing multi-organ injury [78]. SARS-CoV-2 infection can cause the disruption of many pathways that use ACE2 for proper functioning, and may result in injury and impairment, including cognitive impairment [77]. SARS-CoV-2 has high neurotropism for the CNS, and ACE-2 receptor expression has recently been found on neurons and glial cells of several brain structures, including the cerebral cortex, striatum, substantia nigra and brain stem [79]. The mechanisms of invasion by SARS-CoV-2 are threefold, i.e. via the olfactory bulb (from the olfactory epithelium into brain regions receiving olfactory projections), via the hematogenous route (attaching to the ACE2 receptor expressed in endothelial cells of cerebral blood vessels, or inside immune cells), or via nerve terminals of the vagus nerve, in the respiratory system or gastrointestinal tract for example [79, 80]. These mechanisms promote entry of the virus into the brain, and during the acute phase of COVID, may give rise to a range of neurological symptoms, including (but not limited to) encephalitis and meningitis, anosmia, ageusia, or Guillain-Barré syndrome [81].

During the acute phase of COVID-19 infection, the respiratory system is primarily affected, whereas in the longer term, there are manifestations in other organs and systems. Definitions of these persistent infectious periods distinguish between the acute phase (up to 4 weeks), post-acute (4–12 weeks), long post-COVID (12–24 weeks) and persistent post-COVID (> 24 weeks) [82]. It is important to note that post-COVID is a syndrome, not a disease, because its pathogenesis is unifactorial, and characterized by the infection, but the clinical manifestations are pleiotropic with many systems involved. Furthermore, it impacts on functional status (causing disability). The prevalence of “long” COVID is high around the world, and a systematic review from 2021 estimated that in Italy, 25% of patients have persisting symptoms at 12 weeks after infection [83].

Risk factors for long COVID include abnormal laboratory results, underlying comorbidities (e.g. diabetes, hypertension, cardiovascular diseases), severity of initial disease (need for intensive care, ventilatory assistance), and importantly, older age [84]. Several age-related endocrine and immunologic factors predispose older individuals to being disproportionately affected by long-COVID. These include cellular and immuno-senescence, inflammaging, lower levels of physical activity, undernutrition, and more frequent comorbidities [85]. Importantly, the general symptoms in older individuals frequently feature cognitive impairment, the mechanisms of which are multiple. First and foremost, respiratory system inflammation can cause neuroinflammation and microglial activation, leading to neuronal and glial dysregulation, together impairing the function of neural circuits, and giving rise to the characteristic “brain fog”. Second, COVID-19 infection may trigger reactivation of latent herpesviruses, compounding the inflammation. Third, there may be disruption of the integrity of the blood–brain-barrier, causing neuroinflammation and injury [85]. A recent retrospective cohort study in over 6.2 million adults aged 65 years and over found that people with COVID-19 had a significantly increased risk of new onset AD within a year after the initial COVID-19 diagnosis (hazard ratio HR:1.69, 95% CI 1.53–1.72) [86, 87].

The dementia process involves protein aggregation and toxicity to neurons, promoting synapse dysfunction and ultimately, clinical manifestation of dementia [88]. SARS-CoV-2 accelerates these processes [89, 90]. Interesting it has also been shown that SARS-CoV-2 promotes the probability of aggregation of misfolded proteins, for several reasons due to virus structure (spikes) and cleavage processes. When the proteases promote cleavage to allow the virus to enter the cell, the misfolded proteins start to accumulate on the surface of the virus, and the cleavage of the virus itself leads to release of peptides that have high aggregation propensity. This has been demonstrated in animal models and in vitro, showing that SARS-CoV-2 infection exacerbates alpha-synuclein misfolding, aggregation and interruption with alpha-synuclein clearance [90]. In AD brains, COVID infection may accelerate accumulation of beta amyloid and formation of fibrils. Deposits increase, and clearance is reduced, resulting in faster progression of disease in those with AD, and higher rates of entry into the disability phase. From a functional point of view, this has many consequences. In one study of PET scans from 35 patients with long COVID compared to 44 healthy subjects, those with long COVID had lower metabolism in various brain systems and functions. Specifically, those with long COVID exhibited bilateral hypometabolism in structures involved in attention, short term memory and visuo-spatial ability (rectal/orbital gyrus, olfactory gyrus, right temporal lobe) [91]. A second study in 785 participants aged 51–81 years in the UK biobank found a greater reduction in grey matter thickness and tissue contrast in the orbitofrontal cortex and parahippocampal gyrus, as well as a greater reduction in global brain size in SARS-CoV-2 cases [92]. The authors also showed in a functional evaluation that cases affected with COVID had lower performance on tests of attention, spatial ability and short-term memory, indicating that not only is infection associated with neurodegeneration and structural changes in the brain, but also with functional change in cognitive performance [92].

In summary, after onset of infection with SARS-CoV-2, there may be persistence of symptoms related to two main mechanisms: first, SARS-CoV-2 promotes aggregation of misfolded proteins in the neurons, and the brain by various direct and indirect mechanisms, leading to neurodegeneration and cognitive dysfunction. Second, via circulatory mechanisms, the impact of the virus on lung function and hypoxia together promote neurodegeneration and cognitive decline.

### HIV-associated neurocognitive disorders

The latest epidemiological data suggest that there were 38.4 million people living with HIV (PLHIV) in the world in 2021, with 1.5 million newly infected and 650,000 deaths from AIDS-related illness in the same year [93]. The numbers of PLHIV accessing treatment have improved significantly over the last 20 years, and as of 31 December 2021, 28.7 million people were receiving antiretroviral therapy (representing 75% of all PLHIV) and up from 7.8 million in 2010 [93].

In the early HIV era (before the introduction of highly active antiretroviral therapy (HAART), up to 15% of patients were diagnosed with AIDS dementia complex, and this was associated with large ventricles, atrophy and significant white matter involvement. Patients tended to be young, developing dementia within 5–10 years of infection [94]. While the symptoms were partially reversible with HAART, there was no complete cure, and patients usually had typical computed tomography (CT) and magnetic resonance imaging (MRI) changes that included CNS opportunistic disorders. Nowadays, neurocognitive disorders observed in PLHIV are termed HIV-associated neurocognitive disorders (HAND). The diagnosis is made mostly by exclusion, namely there should be no delirium, no other pre-existing cause, no untreated depression, no active substance abuse. It corresponds to acquired impairment in at least two ability domains on cognitive functioning tests of at least 5 or 6 domains [95, 96]. The prevalence of HAND is between 30 and 60%, but differs according to the tests and criteria used, and according to the burden of comorbidities that may affect the CNS [97]. In Europe, many PLHIV have asymptomatic impairment, but mild impairment [98, 99] may be associated with lower adherence to drugs, a higher risk of failing antiviral regimens, and progression over time [98, 99].

In terms of pathogenesis, HIV reaches the CSF and the brain as early as day 6 after infection [100], usually brought there by infected CD4 cells, since cell trafficking is important for establishing and maintaining a HIV reservoir in the brain. Of note, HIV cannot affect neurons, as neurons lack both CD4 and CCR5 receptors, but it can infect the microglia, astrocytes (at least partially), and oligodendrocytes. This generates long-term low-grade inflammation, producing neurotoxic products that damage neurons. There is also a potential effect of HIV proteins, which continue to be produced despite antiretroviral treatment: for example, the Tat protein is known to directly cause neuronal damage and to increase the risk of amyloid deposition inside neurons [101]. Significant amyloid deposition has been demonstrated in the brain of PLHIV with a direct correlation between years of infection and the pathological extension [102, 103]. However, it remains to be determined whether HAND leads to AD, or whether it is a specific form of virus-associated dementia [104].

Considering the direct role of the virus, HIV RNA can be detected in the CSF of the majority of seropositive patients [105], with cART being effective in reducing HIV RNA levels to undetectable levels [106]. However, in some patients, there may be viral escape, whereby the virus can be detected in the CSF even though there is no detectable viremia. It remains unclear whether low-level viral replication in the CSF can be associated with neurocognitive damage over time [107].

A challenge in the drug therapy of PLHIV is achieving a balance between neuroefficacy and neurotoxicity. There are various mechanisms of neurotoxicity, including direct neuronal toxicity, interference with beta amyloid metabolism, or effects on astrocytes and the blood–brain barrier. An autopsy study from the US showed that tenofovir use was associated with lower odds of amyloid beta deposition, while darunavir use prior to death was associated with higher odds of phosphorylated tau deposition in neurons, and ritonavir use prior to death was associated with higher odds of microgliosis [108].

In PLHIV, the blood–brain barrier is impaired very early in the course of HIV due to the infection. The blood–brain barrier has been shown to play an important role in the pathogenesis and development of HAND, with 68–100% of individuals with HAND having signs of blood brain barrier impairment. Its pathogenesis is multifactorial, including pro-inflammatory cytokines and viral proteins from infected cells, and direct invasion of pericytes and perivascular macrophages by HIV [109, 110]. Up to 30% of neuro-asymptomatic PLHIV have blood brain barrier impairment, but there is conflicting evidence regarding the effect of antiretroviral therapy on blood brain barrier integrity [111, 112].

There are a number of features that distinguish HIV-associated dementia from Alzheimer’s dementia, notably regarding the relationship between amyloid and tau proteins, which seems to differ in PLHIV compared to AD subjects. In a case series of four seropositive patients diagnosed with AD, who were all on highly active antiretroviral therapy with non-detectable serum HIV RNA, Calcagno et al. established patterns of patients according to biomarkers, and found that the biomarker patterns were associated with cognitive functions [113]. In another retrospective study of adult PLHIV (on cART with undetectable viremia, *n* = 136, with detectable viremia, *n* = 121, and with CNS disorders regardless of viremia, *n* = 72; and HIV-negative controls with AD, *n* = 84), Trunfio et al. reported that the majority of participants (79.6%) presented normal CSF AD biomarkers, and no HIV-specific features were associated with canonical CSF AD biomarkers [114].

In summary, mild cognitive impairment is prevalent even in PLHIV with effective cART. There is a complex pathogenesis, with direct and indirect neuronal damage, caused not only by the infection but also by the drugs used to treat HIV. Older PLHIV at high risk of dementia need to be screened and studied over time.

### Herpes simplex virus type 1 (HSV-1)

After primary infection, herpes viruses can remain latent in the body, and can be reactivated later by stress, immunosuppression, or inflammation. Reactivation of the virus, denoted by productive infection, causes direct viral damage and inflammation, and recurrent events over time probably cause cumulative damage. With the advent of polymerase chain reaction (PCR) techniques in the 1980s came the first demonstration that HSV-1 DNA could be detected in the brain of patients with AD. Later studies expanding on these findings further showed the presence of anti-HSV-1 antibodies in the CSF, which can persist for years after herpes simplex encephalitis (HSE), suggesting that the virus was latent and could reactivate. Detection of these HSV-1 antibodies in both AD and normal aged subjects, shown not to be due to leakage across the blood-CSF barrier, indicated that HSV-1 DNA resides in many elderly brains and may have been reactivated, perhaps recurrently, potentially causing acute infection.

It is now established that the combination of HSV1 DNA in the brain and the type 4 allele of the gene for apolipoprotein E (APOEe4, a known susceptibility factor for AD) confers a strong risk of AD [115].

HSV-1 infection produces amyloid beta and abnormally phosphorylated tau [115, 116]. It is likely that carriage of the type 4 allele of the gene for apolipoprotein E (APOEe4) together with the presence of HSV-1 confers the damage, likely through repeated reactivation through immunosuppression. In a study using PCR to detect HSV-1 DNA, and immunohistochemistry or thioflavin S staining to detect amyloid plaques, Wozniak et al. showed that there was exact superposition of the HSV1 DNA on the localization of amyloid beta plaques in AD brains, suggesting that HSV-1 viral DNA was perhaps encaged in the amyloid plaques [117]. Subsequent investigations found that administration of anti-HSV1 antiviral agents, notably acyclovir, decreased amyloid beta deposition and inhibited HSV-1-induced abnormal tau phosphorylation [118].

Against this background, the so-called HSV1-AD concept was formulated, hypothesizing that HSV1 resides latently in brain of many elderly people but can reactivate, probably repeatedly, as shown by the presence of antibodies in the CSF. On repeated reactivation, HSV-1 causes limited and localised but cumulative damage that may eventually lead to AD. There is a solid body of evidence in support of this, notably showing that HSV-1 antibodies are present in CSF of AD patients and age-matched normal elders, indicating that HSV-1 replication and expression both occur in brain [115]. HSV-1 DNA is detected in the CSF of a much higher number of people than would be expected, if it occurred only after Herpes simplex encephalitis, which is a very rare disease, thus suggesting that virus reactivation is frequent [119, 120]. Furthermore, HSV-1 DNA is detected in brain of immunosuppressed and HSV-seropositive patients, but not in immunosuppressed HSV-seronegative patients or in immunocompetent individuals [121].

There is also a large body of evidence in favour of the involvement of HSV-1 in AD. Reactivation of HSV seropositivity (IgM) was shown to be associated with the risk of AD in longitudinal, population-based studies of community-dwellers [122, 123]. As mentioned above, HSV-1 is known to cause accumulation of amyloid beta with HSV-1 DNA located specifically in AD plaques [116, 117]. HSV-1 also causes AD-like accumulation of hyperphosphorylated tau [124]. In a 3D cell culture brain model, VZV infection reactivates latent HSV1 [125], consistent with the known increase in risk of AD caused by infections, and consistent with the suggestion that vaccination decreases risk of AD by reducing infections, thereby reducing reactivations of HSV1 in brain [126]. In this regard, vaccination against shingles was found to reduce the risk of AD [127]. In animal models, repeated reactivation of HSV-1 in brain of infected mice was found to cause an AD-like phenotype and cognitive decline [128]. Population-level epidemiological data from Taiwan reported that HSV infection was associated with a 2.56-fold increase in the risk of developing dementia, whereas a significant risk reduction for dementia was observed in patients affected by HSV infections and treated with anti-herpetic medications (adjusted hazard ratio 0.092 [95% CI 0.079–0.108], *P* < 0.001) [129, 130]. Taken together, the current body of findings in the literature plead in favour not only of the involvement of HSV-1, but of a causal role for HSV-1 in AD.

### Influenza and dementia

Influenza virus is another pathogen that has been shown to be capable of entering the CNS. Indeed, it has been shown in murine models that the H5N1 influenza virus can travel from the peripheral nervous system into the CNS, and in the regions infected by H5N1 virus, there was activation of microglia and alpha-synuclein phosphorylation and aggregation, which persisted even after the infection had resolved [131]. These authors concluded that a pandemic H5N1 pathogen, or other neurotropic influenza virus could be capable of initiating CNS disorders that lead to protein aggregation, such as PD or AD. Other authors subsequently expanded on these findings, and showed that influenza has effects on hippocampal dendrites and on inflammation, suggesting that neuroinflammation and changes in hippocampal structural plasticity may underpin the cognitive dysfunction associated with influenza infection [132].

Translating these pre-clinical findings into humans, there is a rationale to hypothesize that influenza infection could be implicated in the pathogenesis of AD. Indeed, there is evidence attesting to the fact that influenza is associated with neuropsychiatric disorders, including confusion, delirium, convulsions, and encephalopathy [133]. Evidence further suggests that the neurological impact of influenza is due to neuroinflammatory insult, which is largely immune mediated, as opposed to the result of direct viral invasion of the CNS [132]. However, the literature about the risk dementia after influenza is overall negative [134].

Several reasons may explain why influenza has not been found to have a significant association with dementia in humans. Firstly, influenza is a clinical diagnosis that is usually made based on clinical presentation, and it is rarely supported by viral tests. Thus, one cannot rule out the possibility that some recorded influenza diagnoses were not caused by an influenza virus. Second, influenza is a disease that may quite easily be self-treated, and therefore, not all patients with the illness come to the attention of healthcare providers. Other methodological issues include the relatively short follow-up of studies to date. At the same time, it has been confirmed that past exposure to vaccines against diphtheria or tetanus, poliomyelitis and influenza may protect against subsequent development of AD [135]. Other authors have also shown that influenza vaccination reduces dementia risk in chronic kidney disease patients in a cohort of 11,943 patients from the National Health Insurance Research Database of Taiwan [136]. In this study, there was not only an overall significant protection of influenza vaccination against dementia, but the effect was more evident in those aged > 70 years, and vaccination dose dependently reduced the risk of dementia in all subgroups [136]. Similar results were reported by Luo et al. in a nationwide retrospective cohort study (also from Taiwan) of 19,848 patients aged over 60 years with chronic obstructive pulmonary disease (COPD) [137]. In that study, there was a statistically significant and clinically substantial decrease in the risk of dementia in vaccinated vs unvaccinated COPD (aHR for dementia 0.68 (95% CI 0.62–0.74, *P* < 0.001 for vaccinated vs unvaccinated patients), and a dose-dependent effect, as observed in earlier studies.

A systematic review and meta-analysis of recent epidemiological evidence was published in 2022, confirming that influenza vaccination was associated with a significantly lower risk of dementia in older individuals [138]. Another recent study used propensity score-matching to produce a sample of 935,887 vaccinated vs unvaccinated matched pairs from among an unmatched sample of 2.3 million eligible patients [139]. The authors reported that influenza vaccination was associated with significantly reduced AD risk in this sample of US adults aged 65 and older, corresponding to a number needed to treat of 29.4, suggesting that influenza vaccination could be a simple and inexpensive intervention to prevent dementia.

The mechanisms by which influenza vaccination could prevent dement are likely mediated by neuroinflammation. In animal models, it was shown that early influenza vaccination activated microglia, reduced the amyloid beta burden and improved cognitive impairment [140]. The action against neuroinflammation and the improvement of neuroplasticity shown in this study could at least partially explain how vaccination affects AD risk. Furthermore, there is likely a pleiotropic effect of influenza vaccination regarding other outcomes, e.g., CV disease incidence, whereby a decrease in vascular events at cerebral and non-cerebral level could also decrease the risk of dementia. Evidence in lowering the risk of death in community-dwellers older people, of all deaths/severe respiratory diseases in high-risk community-dwellers and of hospitalization for influenza/pneumonia in case–control studies, was highly suggestive [141]. Indeed, Govindpani et al. again highlighted that the microglia and blood brain barrier disruption are probably key factors since vascular impairment occurs and worsens at every stage of the dementia disease process. By modulating these factors, influenza vaccination could therefore have a protective effect against dementia.

In conclusion, despite significant research efforts, there is still a lack of efficacious interventions that can offer long-term symptomatic relief from dementia. The available literature reported a reduced risk of dementia in selected populations, including patients diagnosed with various medical conditions such as chronic kidney disease and COPD following administration of the influenza vaccination. Influenza vaccination could theoretically offer an inexpensive, low-risk mechanism of prevention of dementia.

## Conclusion

Over the last few decades, there has been outstanding progress in our understanding of the role of microorganisms in neuroinflammation and dementia. However, encouraging pre-clinical findings have not translated into similarly impactful findings in human studies. Further research is warranted in several areas, notably the field of biomarkers, to identify early-stage disturbances of homeostasis in the brain and CNS; and in longitudinal studies, to investigate the long-term effects of infection, whose harmful effects may take years, not to say decades to become apparent. The interactions between individuals, genetics, environmental and lifestyle factors, microbes, and immune response are extraordinarily complex. Just as there is wide heterogeneity in the process of aging between individuals, so there is similar variety in the individual response to infection. Going forward, we need to be mindful that there may be a time lapse of years, or even decades between a primary infection and later onset of cognitive decline. Tracking the processes at work during the whole period in between should be the objective of future studies. Identification of early inflammatory markers, biomarkers of dementia, and long-term follow-up of gut microbiota studies are needed to help monitor progression from infection to cognitive dysfunction. A particularly attractive preventive option is the wider use of vaccines to prevent infection, thereby mitigating the possible injurious effects of infection on the brain, with the potential for cognitive decline. The departure point (infection) and the arrival point (dementia) are both known, but the road from one to the other is long, and remains largely uncharted territory waiting to be explored.

## Data Availability

Data sharing not applicable to this article as no datasets were generated or analysed in the present work.
